# JOURNAL OF GERONTOLOGY PSYCHOLOGICAL SCIENCES: FEATURED 2023 EDITOR’S CHOICE ARTICLES

**DOI:** 10.1093/geroni/igae098.1348

**Published:** 2024-12-31

**Authors:** Duke Han

**Affiliations:** Chair

## Abstract

The psychological sciences of the aging process are vast in scope and complexity. Methodological approaches that are interdisciplinary, innovative, and inclusive can make these enigmatic processes more tangible. This symposium showcases articles in the Journal of Gerontology Psychological Sciences that were selected as Editor’s Choice in 2023 because they exemplify these ideals. Smith et al. investigate cognitive ability differences by in-person versus telephone-based assessment methods. Jackson et al. explore associations between loneliness and cognitive resilience to brain neuropathology in older adults. Krok-Schoen et al. report on the resiliency of women aged 80 and older by race, ethnicity, and neighborhood socioeconomic status. Esiaka and Luth describe different interpretations of “honoring your parents” in the U.S., Ghana, and Nigeria. Lynch et al. link measures of loneliness, DNA methylation, and cognitive functioning.

